# Surface Complexation Models of Pertechnetate on Biochar/Montmorillonite Composite—Batch and Dynamic Sorption Study

**DOI:** 10.3390/ma13143108

**Published:** 2020-07-12

**Authors:** Martin Daňo, Eva Viglašová, Michal Galamboš, Karel Štamberg, Jan Kujan

**Affiliations:** 1 Department of Nuclear Chemistry, Faculty of Nuclear Sciences and Physical Engineering, Czech Technical University in Prague, Břehová 7, 115 19 Prague, Czech Republic; karel.stamberg@fjfi.cvut.cz (K.Š.); kujanjan@fjfi.cvut.cz (J.K.); 2 Department of Nuclear Chemistry, Faculty of Natural Sciences, Comenius University in Bratislava, Mlynska Dolina, Ilkovicova 6, 842 15 Bratislava, Slovakia; michal.galambos@uniba.sk

**Keywords:** engineered biochar, XRF, potentiometric titration, technetium, rhenium, sorption, equilibrium isotherm, pH dependencies, column experiments, mathematical modeling

## Abstract

The study summarizes the results of monitoring the properties of two types of sorbents, BC1 (biochar sample 1) and BC2a (biochar sample 2), prepared by pyrolysis of bamboo biomass (BC1) and as its composite with montmorillonite K10 (BC2a). The main goal was to study their applicability to the Tc (VII) separation from liquid wastes, using NH_4_ReO_4_ as a carrier. The research was focused on determining the sorbents surface properties (by XRF (X-ray fluorescence) method and potentiometric titration in order to determine the properties of surface groups—Chemical Equilibrium Model (CEM) and Ion Exchange Model (IExM) models were applied here). As well as monitoring Tc (VII) (+Re(VII)) sorption, especially to determine equilibrium isotherm, the influence of pH and kinetics. The subject of research was also the dynamics of sorption, including its mathematical–physical modeling. Both sorbents have good properties against Tc (VII), however BC2a, due to the presence of montmorillonite, is more advantageous in this respect. It has a higher sorption capacity and faster kinetic investigation. An important finding is that the optimal pH is 2–3, which is related not only to the protonation of surface groups (they have a positive charge), but also to the negative form of the existence of Tc (VII) and Re (VII): TcO_4_^−^ and ReO_4_^−^.

## 1. Introduction

The fission product, a long-lived, low-energy beta emitter and medical radioisotope, technetium-99 (^99^Tc), is generated in the course of nuclear reactor operation. It is the main component of nuclear fuel waste driving from the fission of ^235^U and ^239^Pu and also a common contaminant in the subsurface at nuclear facilities. Into the environment, the technetium has been accidentally introduced by the waste storage facilities leaks and currently is a key risk driver [1,2]. Technetium occurs in various oxidation states based on the environmental redox condition. The pertechnetate anion TcO_4_^−^, oxidation state VII+ is the most common chemical form of technetium, mainly in liquid nuclear wastes and the environment [1,3]. Chemical and physical properties of technetium are common with rhenium. The nature of noncomplexing, high water solubility, high volatility and negative charge of pertechnetate, all take responsibilities for its high mobility in water, making it a problematic nuclide during the environmental restoration activities [4]. The novel and practical technologies for pertechnetate sequestration/removal, are needed to reduce the potential contamination of the environment and the threat to living organisms [1]. Therefore, developing high-efficient functional materials that can rapidly and selectively capture TcO_4_^−^, is of great significance [4].

There are currently several general approaches for TcO_4_^−^ immobilization from waste. However, the amount of environmental ^99^Tc is normally very small, a preconcentration of ^99^Tc is usually required. Environmentally occurring aqueous samples (e.g., rainwater, water from lakes and rivers, groundwater, etc.) should be pretreated in the first step. This includes filtration through a suitable filter to remove suspended particles, acidification to pH 1–4 with HCl [5], H_2_SO_4_ [6] or HNO_3_ [7]. Above mentioned technetium immobilization may be achieved by several methods, such as evaporation [8], extraction chromatographic methods [9], sorption onto carbon-based materials such as biochar [10], etc.

The evaporation technique is a very simple method and is applicable to smaller volumes of samples with low salinity, respectively. ^99^Tc is a volatile element, therefore evaporation takes place at a temperature below 90 °C. Larger losses occur by evaporation to dryness, so it is advantageous to evaporate samples to almost dryness [11]. Based on it, this technique is not practical for large sample volumes and high salinity samples. Therefore, the use of other preconcentration techniques is more advantageous.

An example of the extraction chromatographic technique is the use of TEVA^®^ Resin. The resin that contains the quaternary ammonium functional groups of Aliquat^®^ 336, the alkyls of these groups forming a mixture of octyl and decyl chains. This material is most often used for the separation of tetravalent actinides and technetium (VII) [9]. The identification and quantification of ^99^Tc by accelerator mass spectroscopy (AMS) face not only (albeit in relatively small amounts) the nuclide isobar ^99^Ru, but the issue also is the lack of a stable technetium isotope [12]. Therefore, a key step for the preparation of technetium matrices, and subsequent AMS measurements, is the rapid separation of technetium from interferents, because it is the conversion of technetium that produces the ruthenium isobar [13]. In addition, when the amount of ^99^Ru in the sample is as small as possible, AMS detectors are able to determine ^99^Tc from ^99^Ru [14]. In addition to water samples, TEVA^®^ Resin allows the separation of technetium from soil, sediment and urine matrices [15]. The highest achievable values of technetium retention factors for this material are approximately 5·10^4^. Separation processes of this extraction chromatographic material are fast, more efficient than liquid extraction or precipitation techniques. They include oxidation of Tc (IV) to Tc (VII), oxidation of organic impurities with H_2_O_2_, and selective elution with nitric acid solutions [16].

Sorbents based on carbon, such as biochar, are effective sorbents for technetium removal. Their advantages are in the large specific surface area, porous structure, content of noncarbonized fractions (noncarbonized organic matter) and thus high variability in surface functional groups. Various cationic forms of radionuclides have been shown to be successfully removable by biochar-based sorbents based on their negative surface charge. However, most of the traditionally produced have limited ability to sorb anionic species. Recently a new concept, engineered biochar has gained interest, in which engineered biochar is prepared by biochar surface impregnation with montmorillonite or other clay minerals [3,17,18,19,20]. Therefore, technetium in the most environmentally stable form—pertechnetate is sorbed, while others, mostly metals flow through this material if pH is smaller than approx. 2–3. This is particularly advantageous for large sample volumes circulating through a column packed with these kinds of materials [1,20]. Additionally, application of engineered biochar as a low cost and more environmentally friendly method in environmental applications compared to conventional technologies [such as precipitation, filtration or coagulation represent a future step firstly, in assessment and recycling of waste materials in 21st century, secondly for technetium separation application [2,3,17,21].

In our previous work, it was shown the fundamental understanding of the changes in the biochar structure as a function of mineral additives is therefore crucial for the implementation of strategies to design biochar with superior properties, tailored to enhance performance. For this purpose, the biochar/montmorillonite composite was a detailed analysis of the structure. Prepared and characterized materials were tested for sorption studies of nitrate from aqueous solutions [17]. The objectives of this study were to enrich and supplement the characterization of prepared material structural properties; investigation of batch sorption experiments (pH, Contact Time, Equilibrium Study); dynamic sorption experiments (Breakthrough Sorption Curves); and surface processes calculations based on Acid–Base Titrations, in order to improve the environmental applicability of prepared engineered biochar composite for technetium immobilization and determination.

## 2. Materials and Methods

### 2.1. Materials Preparation

All biochar samples from raw bamboo biomass were prepared by pyrolysis at 460 °C in a furnace with a residence time of 120 min. The nitrogen (N_2_) was used as flush gas, to uniform heating conditions and to ensure an oxygen-free environment. After pyrolysis, prepared materials were rinsed several times with deionized water and oven-dried at 80 °C for 24 h. Samples were ground, sieved (particle size of 0.5–1 mm) and stored in boxes till the next use. Raw biochar sample was labeled as sample BC1. The biochar/montmorillonite composite (sample labeled BC2a) was prepared from a mixture of bamboo biomass pretreated with montmorillonite K10 (MK10; chemical formula (Na,Ca)_0.33_(Al,Mg)_2_(Si_4_O_10_)(OH)_2_·nH_2_O)). Sample BC2b was prepared from BC2a (see below). The samples’ preparation in detail was well-described in our previous work [17]. All chemicals and solvents used during experiments were of analytical grade quality.

### 2.2. Materials Characterization

The characterizations (surface area determination by nitrogen adsorption (NOVA 1200e Surface Area & Pore Size Analyzer, Quantachrome Instruments, Boyton Beach, FL, USA), field emission scanning electron microscopy (TESCAN MIRA 3, Oxford Instruments, Abingdon, UK), and Fourier-transform infrared spectroscopy (VERTEX 70, Bruker UK Ltd., Durham, UK)) were performed for both prepared materials BC1 and BC2a and they were well characterized in our previous work [17]. In order to complete and improve their surface properties, the X-ray fluorescence (XRF) and potentiometric, acid–base titrations [22] were performed.

The XRF spectrometer NITON XL3t 900Analyzer with GOLDD Technology (Thermo Scientific, Waltham, MA, USA) was used as a nondestructive qualitative technique, the simplest and most accurate analytical technique for a samples’ surface elemental characterization. Before performing the analysis, the NITON was allowed to warm up for a minimum of 15 min. Prior to measurements, the spectrometer was calibrated. The 4 μm thin film (3252 ULTRALANE^®^, Spex SamplePrep, Metuchen, NJ, USA) was stretched at one end of the double-opened ring cup (SC-4331, Premier Lab Supply, Lucie, FL, USA) and attached with an oversize ring. Samples were sprinkled into these cups. Cups with samples were placed over the detector and measured in the “mining Cu/Zn” mode.

Acid–base titrations curves were studied with respect to basic parameters of the surface sites. To remove carbonates from the montmorillonite part of sample BC2a, the procedure on this sample described in [23] was performed before titration experiments. The following solutions were prepared. Solution A: 1 mol·dm^−3^ NaNO_3_ (Penta s.r.o, Prague, Czech Republic) + 0.001 mol·dm^−3^ HNO_3_ (Lach-Ner Ltd., Neratovice, Czech Republic). Solution B: 0.1 mol·dm^−3^ NaNO_3_ (Penta s.r.o., Prague, Czech Republic). In each of two 50 mL PP centrifugal test tubes (P-Lab a.s., Prague, Czech Republic), 1 g of BC2a was mixed with 40 mL of solution A, 20 min at 350 rpm (IKA Labortechnik KS250 basic shaker, IKA^®^-Werke GmbH & Co. KG, Staufen, Germany). The phases were separated through the filter paper (Whatman, grade 43). Phases mixing and their separation on was repeated until a stable pH 3 of the filtrate was reached. Then, the content of both centrifugal test tubes was transferred to an Erlenmeyer flask. A 100 mL volume of solution B was added, and suspension was shaking for 10 min at 350 rpm repeatedly after separation on filter paper. The pH value of the filtrate stabilized at 6.6. The resulting sample labeled as BC2b was dried at 90 °C overnight. Sample BC1 did not require any post-treatment.

An amount of 0.2 g of each sample was suspended in 50 mL, 0.1 mol·dm^−3^ NaNO_3_ (Lach-Ner Ltd., Neratovice, Czech Republic), to keep up ionic strength constant in a PE container. This container was placed in the TitraLab^®^ 845 titration (Hach Ireland, Little Island, Ireland) workstation with a TIM845 titration manager. Prior to each titration suspension was stirred for a minimum of 30 min to establish equilibrium between solid and aqueous phase. Two separate branches were carried out, the acidic (titration with 0.1 mol·dm^−3^ HNO_3_, (Lach-Ner Ltd., Neratovice, Czech Republic)) which ended under pH equal to 3, and basic (titration with 0.1 mol·dm^−3^ NaOH, (Lach-Ner Ltd., Neratovice, Czech Republic)), which ended at pH 10. pH was measured by PHC2401-8 combination red-rod pH electrode (Radiometer Analytical, city, France). In the course of titrations stirred suspensions were bubbled to keep systems under N_2_ atmosphere. The total titration time was approx. 8 h.

The modeling of titration curves is described in detail in [22,24]. It is assumed that there are two types of surface, functional groups, namely the so-called edge and layer sites. These have different properties, so three types of surface complexation models (SCM) are used to describe edge sites, namely two electrostatic (CCM—Constant Capacitance Model and DLM—Diffusion Double Layer Model) and one without electrostatic correction (CEM—Chemical Equilibrium Model). Processes running on layer sites are described using the IExM—Ion Exchange Model.

In general, if CEM and IExM models are taken into account [22], reactions taking place on the surface of biochar can be described by Equations (1)–(3):

≡SO⁻ + H⁺ ↔ ≡SOH^0^ (1)

≡SOH^0^ + H^+^ ↔ ≡SOH_2_^+^ (2)

≡XNa + H^+^ ↔ ≡XH + Na^+^ (3)

where ≡SO^−^, ≡SOH^0^ and ≡SOH_2_^+^ are symbols for edge sites, X^−^ is the symbol for layer sites. The equilibrium constants *K*_1_, *K*_2_ and *K_ex_* are given by Equations (4)–(6):

(4) $$K_{1}=\frac{\left[{\mathrm{SOH}}^{0}\right]}{\left[{\mathrm{SO}}^{-}\right]\left[H^{+}\right]}$$

(5) $$K_{2}=\frac{\left[{\mathrm{SOH}}_{2}^{+}\right]}{\left[{\mathrm{SOH}}^{0}\right]\left[H^{+}\right]}$$

(6) $$K_{ex}=\frac{\left[\mathrm{XH}\right]\left[{\mathrm{Na}}^{+}\right]}{\left[\mathrm{XNa}\right]\left[H^{+}\right]}$$

Surface charge density reaction balances have to be taken into account. Edge sides (7) and layer sides (8) are as follows:

(7) $$\sum \mathrm{SOH}=\left[{\mathrm{SOH}}^{0}\right]+\left[{\mathrm{SO}}^{-}\right]+\left[{\mathrm{SOH}}_{2}^{+}\right]$$

(8) $$\sum X=\left[\mathrm{XH}\right]+\left[X^{-}\right]=\left[\mathrm{HX}\right]+\left[\mathrm{XNa}\right]$$

The actual modeling of a titration curve is performed as follows. In the *i*-th point of the titration curve, the total surface charge density, (*Q_cal_*)*_i_*, equals the sum of charge density on the edge sites, (*Q_ES_*)*_i_*, and on the layer sites, (*Q_LS_*)*_i_*. Therefore (*Q_cal_*)*_i_* = (*Q_ES_*)*_i_* + (*Q_LS_*)*_i_*. Charge density is a function of pH. The values of (*Q_ES_*)*_i_* (Equation (9)) and (*Q_LS_*)*_i_* (Equation (10)) can be calculated.

(9) $${\left(Q_{ES}\right)}_{i}=\frac{\sum {\mathrm{SOH}}^{0}\cdot \left(K_{1}\times K_{2}\times {\left[H^{+}\right]}^{2}+1\right)}{K_{1}\times K_{2}\times {\left[H^{+}\right]}^{2}+K_{1}\times \left[H^{+}\right]+1}$$

(10) $${\left(Q_{LS}\right)}_{i}=\frac{\sum X\times \left[{\mathrm{Na}}^{+}\right]}{\left[{\mathrm{Na}}^{+}\right]+K_{\mathrm{ex}}\left[H^{+}\right]}$$

The experimental value of the surface charge for the *i*-th point of the titration curve, (*Q*_ex_)_i_, can be calculated using Equation (11).

(11) $${\left(Q_{exp}\right)}_{i}=\frac{V_{i}\times \left(C_{a,i}-C_{b,i}+{\left[{\mathrm{OH}}^{-}\right]}_{i}-{\left[H^{+}\right]}_{i}\right)}{m_{i}}$$

where *V_i_* is the total volume of liquid phase; *m_i_* is the mass of solid phase; *C*_a,*i*_ and *C*_b,*i*_ are bulk concentrations of acid (i.e., NO_3_^−^) and caustic soda (i.e., Na^+^) in liquid phase, respectively; the values of *C*_a,*i*_ and *C*_b,*i*_ are given by concentrations of acid and base, and by their consumptions in the course of titration.

Multidimensional regression procedure can be used if *K*_1_, *K*_2_, *K*ex, ∑*SOH* and ∑*X* are sought. We used software Famulus 3.5 (Charles University, Prague, Czech Republic) [24] and in-house computer code P46DNLRG.fm using nonlinear multidimensional regression procedure, Gauss–Newton method. As the criterion of goodness-of-fit, WSOS/DF (weighted sum of squares of differences divided by number of degrees of freedom) is used. Its calculation is based on the χ^2^ -test given by Equation (12), and the WSOS/DF is calculated by Equation (13).

(12) $$\chi^{2}=\frac{\sum {\left(SSq\right)}_{i}}{Sq_{i}^{2}}\;i=1,2,\ldots ,n_{p}$$

(13) $$\frac{WSOS}{DF}=\frac{\chi^{2}}{n_{i}}$$

(14) $$n_{i}=n_{p}-n$$

where (*SS*q)*_i_* is the *i*-th square of the deviation of experimental value from calculated one; (*S*q)*_i_* is the relative standard deviation of the *i*-th experimental point; *n_i_* is the number of degrees of freedom calculated by Equation (14); *n_p_* is the number of experimental points and *n* is the number of model parameters sought during the regression procedure. It holds, if WSOS/DF ≤ 20, then there is a good agreement between the experimental and calculated data. Graphs were plotted in Origin 9.5.

### 2.3. Batch Sorption Study

The sorption properties of BC1 and BC2a samples were studied using the batch equilibrium method. In all cases, an aqueous solution of NH_4_ReO_4_ (Sigma-Aldrich, Saint Louis, Germany, ≥99%)) of the given molarity, labeled with the isotope ^99m^Tc, was used. In batch sorption study, first of all, the pH of solutions must be set. pH affects the surface charges, protonation state of functional groups of edge sites and dissociation state of layer sites, chemical speciation and diffusion rate of the solute [25]. Stock solutions of [^99m^Tc] NaTcO_4_ (DRN 4329 Ultra Technekow FM 2.15–43.00 GBq radionuclide generator, Mallinckrodt Medical B.V, Petten, Netherlands) with the volume activity of 1 MBq·mL^−1^ were adjusted to pH 1.0–9.0 by HCl (Lach-Ner Ltd., Neratovice, Czech Republic) and NH_3_ (Penta s.r.o., Prague, Czech Republic). Into the 20 mg of BC1/BC2a (in 2 mL Eppendorf Safe-Lock Tubes), 2 mL of stock solutions were spiked. Mixtures were shaken (IKA Labortechnik KS250 basic shaker, IKA^®^-Werke GmbH & Co. KG, Staufen, Germany) for 250 rpm at (23 ± 1) °C. After 24 h, suspensions were filtered under pressure through a glass microfiber filter Whatman GF/C. Then, 0.5 mL of filtered solution was taken from each tube and measured for 100 s in a well-type NaI(Tl) scintillation detector NKG-314 with a single-channel analyzer spectrometric assembly NV-3120 (all Tesla, Zbidy, Czechoslovakia). Because of the relatively short half-live of ^99m^Tc, samples’ count rates (*n*_cor_, s^−1^) were corrected considering the time difference between the standard and each sample measurement (*t*_cor_, s) as follows:

(15) $$n_{\mathrm{cor}}=\frac{n}{e^{-\lambda \times t_{\mathrm{cor}}}}\;\left(s^{-1}\right)$$

where *n* (s^−1^) is the sample count rate without background counts, *λ* (s^−1^) is a decay constant of ^99m^Tc.

The sorption percentage (*R*) expresses the proportion of the isotope that was sorbed on the surface of the solid phase after equilibration.

(16) $$R=\left(1-\frac{n_{\mathrm{cor}}}{n_{s}}\right)\times 100\;\left(\%\right)$$

where *n_s_* is the count rate of the standard sample (stock solution aliquot).

By comparing the sorption percentage (*R*) of the samples as a function of pH, the contact time influence onto the sorption percentage was subsequently determined. Weight distribution ratios (*D*_g_) were obtained by measuring the amount of Tc taken up by measured weight of BC from a given volume of aqueous solution.

(17) $$D_{g}=\frac{n_{s}-n_{\mathrm{cor}}}{n_{\mathrm{cor}}}\times BF\;\left(\mathrm{mL}\cdot g^{-1}\right)$$

(18) $$BF=\frac{V_{i}}{m_{i}}\;\left(\mathrm{mL}\cdot g^{-1}\right)$$

where BF is batch factor, ratio of liquid phase *V_i_* (mL) and dry mass *m_i_* (g) of BC.

The study of contact time was performed at a pH of 2 and 4 at which the sorption percentages reached the highest values. Stock solutions of [^99m^Tc] NaTcO_4_ (DRN 4329 Ultra Technekow FM 2.15–43.00 GBq radionuclide generator, Mallinckrodt Medical B.V., Petten, Netherlands) with the volume activity of 1 MBq·mL^−1^ with the specific pH value were prepared. Into the 20 mg of BC1 or BC2a (in 2 mL Eppendorf Safe-Lock Tubes), 2 mL of stock solutions were spiked. At the moment of contact between the solid/liquid phase, time began to be tracked. Suspensions were shaken (IKA Labortechnik KS250 basic shaker, IKA^®^-Werke GmbH & Co. KG, Staufen, Germany) at 250 rpm for 1, 10, 20, 40, 60, 120 and 240 min, at the temperature of (23 ± 1) °C. At that specific time, mixtures were filtered under pressure through a glass microfiber filter Whatman GF/C. A 0.5 mL volume of liquid phase was taken and measured for 100 s in a well-type NaI (Tl) scintillation detector NKG-314 with a single-channel analyzer spectrometric assembly NV-3120 (all Tesla, Zbidy, Czechoslovakia,). The data has been processed by Equations (15)–(18).

The dependence of the adsorbed amount of Re (VII) or Tc (VII) on equilibrium concentration was studied too. The observed contact time and pH of the aqueous phase, as written above, were used in this batch study. Into the 2 mL Eppendorf Safe-Lock Tubes, 20 mg of each BC1/BC2a samples were weighted. Stock solutions of (*c_anal_*) 1 × 10^−7^, 5 × 10^−7^, 1 × 10^−6^, 5 × 10^−6^, 1 × 10^−5^, 5 × 10^−5^, 1 × 10^−4^, 5 × 10^−4^, 1 × 10^−3^, 5 × 10^−3^ and 5 × 10^−2^ mol·dm^−3^ of nonisotopic carrier NH_4_ReO_4_ (Sigma-Aldrich, ≥99%) were labeled by [^99m^Tc]NaTcO_4_ (DRN 4329 Ultra Technekow FM 2.15–43.00 GBq radionuclide generator, Mallinckrodt Medical B.V., Petten, Netherlands) with the final volume activity of 1 MBq·mL^−1^. The tubes were shaken in IKA Labortechnik KS250 basic shaker at 250 rpm. Then, mixtures were filtered under pressure through a glass microfiber filter Whatman GF/C. These data were processed by Equations (15)–(18) and relationships (19) and (20). To describe the dependence of the solute amount on its equilibrium concentration, Freundlich adsorption isotherm was used (21).

(19) $$c_{\mathrm{eq}}=c_{\mathrm{anal}}\times \frac{n_{\mathrm{cor}}}{n_{s}}\;\left(\mathrm{mol}\cdot {\mathrm{dm}}^{-1}\right)$$

(20) $$q=D_{g}\times c_{\mathrm{eq}}\;\left(\mathrm{mmol}\cdot g^{-1}\right)$$

(21) $$q=k_{f}\times c^{n_{f}}$$

where *c_anal_* is the analytical concentration of NH_4_ReO_4_ carrier in mol·dm^−3^, *q* is absorbed amount of the metallic anion per adsorbent dry mass in mmol·g^−1^, *k_f_* and *n_f_* are empirical constants.

In all batch experiments the lower detection limit lied below 1%. Standard deviations were calculated by propagation of uncertainty. Graphs were plotted in Origin 9.5.

### 2.4. Dynamic Sorption Study

**Figure 1:**
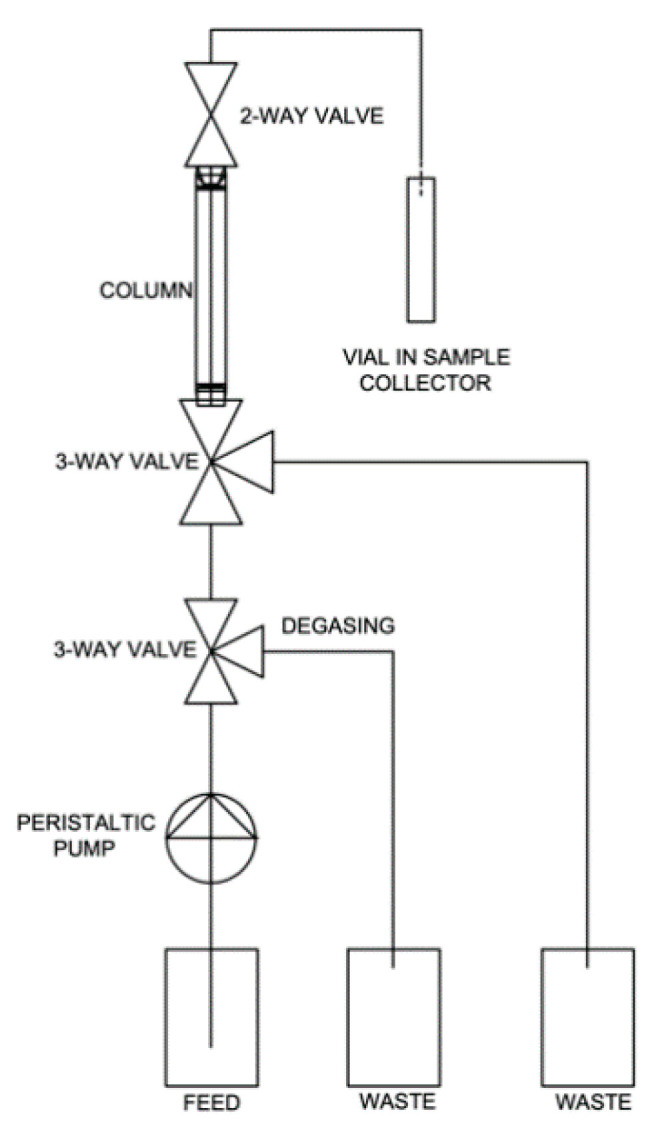
Dynamic sorption layout.

The breakthrough sorption curve is a dependence of relative activity, concentration or count rate of the output solution from column (relative to the feed activity, i.e., to the activity of input sorption solution) on number of bed volume (BV) of output solution. For the dynamic arrangement, the sorption solution consisted of 10^−3^ mol·dm^−3^ (BC1) or 10^−4^ mol·dm^−3^ (BC2a) NH_4_ReO_4_ (Sigma-Aldrich, Saint Louis, MO, USA, ≥99%) water solution as a carrier and labeled by [^99m^Tc] NaTcO_4_. This eluent was adjusted with HCl (Lach-Ner Ltd., Neratovice, Czech Republic) to pH 4 (BC1) and pH 2 (BC2a). A diagram of the installation used in a dynamic adsorption study is shown in Figure 1. The fixed bed adsorptions were carried out in a 1 mL empty Rezorian™ tube kit with PE frits (Supelco, Inc, Bellefonte, PA, USA). Weighted amounts of samples (BC1 0.3437 g; BC2a 0.4310 g) were packed and slightly pressed into these empty columns. Then, they were conditioned with ∼20-bed volumes (BV) of deionized water in an upward flow direction at the temperature of (23 ± 1) °C. The column rested at least for 8 h in order to establish equilibrium between the sorbent and the water. Then, water was drained from the silicon hoses. The column remained water-flooded (1 BV) prior to the sorption study. The linear flow rate was set to ∼0.2 cm·min^−1^ (4.5 BV·h^−1^). Sorption was secured by the peristaltic pump PCD22 (Dávkovací čerpadla Ing. Jindřich Kouřil, Kyjov, Czech Republic), outputs were collected to vials in fraction collector 2210 (Bio-Rad) every 6 min. A 0.5 mL volume of each eluate was transferred to the PE scintillation vial and measured for 100 s in a well-type NaI(Tl) scintillation detector NKG-314 with a single-channel analyzer spectrometric assembly NV-3120 (all Tesla, Zbidy, Czechoslovakia). After ^99m^Tc decay, equilibrium pH was measured by pH meter PHM2200 equipped with a combined pH electrode XC161-9 (Radiometer Analytical, Washington, WA, USA). Graphs were plotted in Origin 9.5.

The transport model of a breakthrough sorption curve is based on erfc function (complementary error function) obtained as a result of the analytical solution of a 1-D ADE (one-dimensional advective dispersion Equation) under sorption boundary conditions [26]. The transport model itself can be modified by incorporation of linear equilibrium isotherm (linear isotherm approach) or nonlinear equilibrium isotherm (nonlinear isotherm approach), which can be found here [27]. The basics of principle are written below. For the theoretical relative output activity of the liquid phase from the column holds:

(22) $${\left(A_{\mathrm{relS}}\right)}_{\mathrm{teor}}=\frac{{\left(A_{\mathrm{tS}}\right)}_{\mathrm{teor}}}{A_{0}}=0.5\times \mathrm{erfc}\left[\frac{{\left(R_{S}\right)}_{\mathrm{teor}}-n_{\mathrm{pvs}}}{2\times \sqrt{\frac{{\left(R_{S}\right)}_{\mathrm{teor}}\times n_{\mathrm{pvs}}}{P_{e}}}}\right]$$

(23) $${\left(A_{\mathrm{relS}}\right)}_{\mathrm{teor}}=\frac{{\left(A_{\mathrm{tS}}\right)}_{\mathrm{teor}}}{A_{0}}=1-\left\{0.5\times \mathrm{erfc}\left[-\frac{{\left(R_{S}\right)}_{\mathrm{teor}}-n_{\mathrm{pvs}}}{2\times \sqrt{\frac{{\left(R_{S}\right)}_{\mathrm{teor}}\times n_{\mathrm{pvs}}}{P_{e}}}}\right]\right\}$$

(24) $${\left(R_{S}\right)}_{\mathrm{teor}}=1+\rho \times \frac{f^{\prime }{\left(C\right)}_{s}}{\varepsilon },f^{\prime }{\left(C\right)}_{s}=\frac{\mathrm{dq}}{\mathrm{dC}}=n_{s}\times k_{s}\times \sqrt[{n_{s}-1}]{{\left(A_{\mathrm{relS}}\right)}_{\mathrm{teor}}\times C_{0}}$$

where: *A_0_*—input activity of liquid phase flowing into the column (cpm), *(A_tS_)_theor_*—theoretical value of liquid phase output activity leaving the column in the course of sorption at time *t_S_* (cpm), *(A_relS_*)*_teor_*—experimental value, *n_PVS_* (= *u*∙*t_S_*/*L*)—experimental value of the number of bed pore volumes in the case of sorption at time *t_S_*, *t_S_*—time of sorption experiment, erfc—complementary error function, *(R_S_)_theor_*—theoretical sorption retardation coefficient, *P_e_* − Peclet number (= *u*∙*L*/*D_d_*) of the column, *u*—liquid phase linear velocity (cm·h^−1^), *L*—bed length in the column (cm), *D_d_*—hydrodynamic dispersion coefficient (cm^2^·h^−1^), *q* and *C*—total concentration of Re(VII) anion in solid (mmol·g^−1^), and in liquid (mmol·mL^−1^) phase, respectively, *C*_0_—starting concentration of Re(VII) in liquid phase (mmol·mL^−1^), *ρ*—bulk density (g·cm^−3^), *ε*—porosity (cm^3^·cm^−3^).

For calculation of *(A_relS_)_theor_*, the Equation (22) can be used directly until *(A_relS_)_theor_* ≤ 0.5, i.e., until (*(R_S_)_theor_* – *n_PVS_*) ≥ 0. For calculation of *(A_relS_)_theor_* > 0.5, i.e., if (*(R_S_)_theor_* − *n_PVS_*) < 0, the Equation (23) should be used, which was derived from Equation (22) (because it holds: erfc *(−x)* = 2 – erfc *(x)*) [28].

## 3. Results and Discussion

### 3.1. Material Characterization

The production conditions strongly affect the physical, chemical and mechanical properties of biochars. A fundamental understanding of the changes in the biochar structure as a function of mineral additives is therefore crucial for the implementation of strategies to design biochar with superior properties, tailored to enhance performance [17]. Concerning the microscopic technique in determining the image and physical morphology conducted by Nartey and Zhao [29], the results of nonbamboo samples are practically the same as for bamboo type BC1. The specific surface area of BC1 (28 m^2^·g^−1^), BC2a (156 m^2^·g^−1^) are comparable with nonbamboo type BC e.g., coconut shells (157 m^2^·g^−1^), orange peel (186 m^2^·g^−1^), sugarcane bagasse (159 m^2^·g^−1^), etc. [30]. FTIR spectra of BC1 and BC2a are similar to nonbamboo types e.g., FTIR spectra of rice (Oryza sativa L) [31], almond shells [32] or corn [33].

In order to determine carbon content in samples, CHN analysis was performed. Carbon content in BC1 is ≥60% (Class 1) and BC2a ≥ 30% (Class 2) of total mass [17] according to the International Biochar Initiative’s standards and classification tool [34]. Thermogravimetric analysis showed that BC1 acted as an efficient carrier of montmorillonite K10 and this symbiosis improved the thermal stability of the bulk composite and can be found in more detail in [17].

#### 3.1.1. XRF

**Figure 2:**
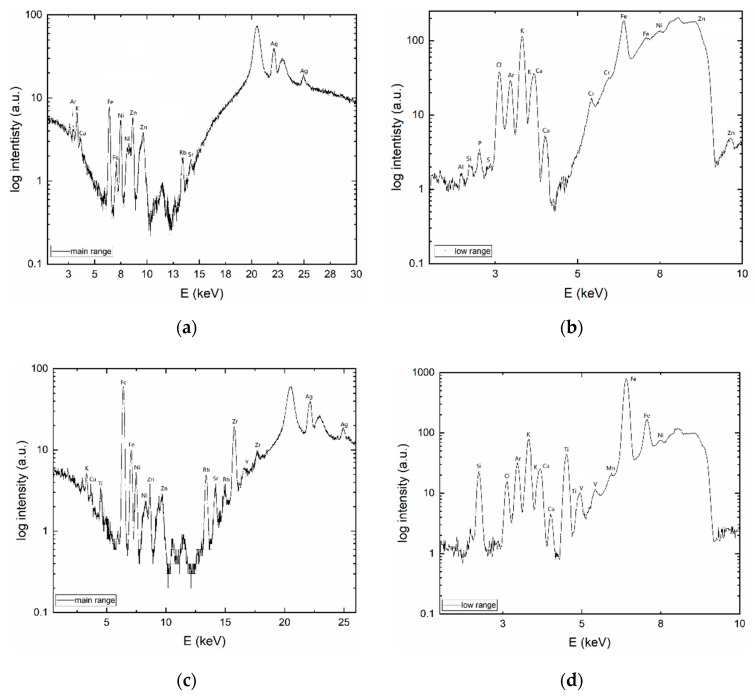
XRF of bamboo biomass (BC1) in (**a**) BC1 main range, (**b**) BC1 low range, (**c**) BC2a main range and (**d**) BC2a low range.

Characteristic emission energies from XRF are shown in Figure 2. Artifact peaks of Fe, Ni, and W, originating from tungsten anode, are in the energy range of (6–11) keV. Only qualitative analysis was carried out. Biochar composition is highly heterogeneous. The major constituents are volatile matter, mineral matter (ash), adsorbed gases, and moisture [35,36]. In BC1, elements Ar, K, Ca, Zn, Rb, Sr and Ag were found. BC2a is a montmorillonite composite, therefore, contains more e.g., Si, and divalent cations in comparison to BC1.

The element identification in XRF spectra is not ambiguous. The overlapping of peaks is one of the disadvantages of XRF. Therefore, identified argon’s K levels can be attributed to L levels of silver atoms.

Data on BC’s XRF, elements heavier than Ca are rather limited. However, it can be found for nonbamboo type BC that XRF qualitative analysis is approximately the same as for BC1 and BC2a. For example, the same elements Mg, Mn, Fe, Zn and Sr were found in wheat and rice straw biochars [37].

#### 3.1.2. Acid–Base Titration

**Figure 3:**
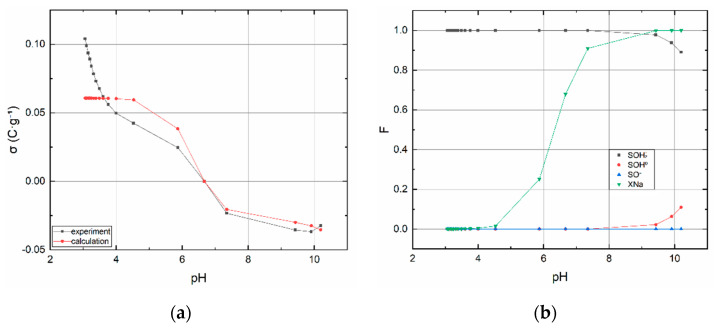
BC1 (**a**) Surface charge dependence on pH and (**b**) species’ mole fraction dependencies on pH. Chemical Equilibrium Model (CEM) and Ion Exchange (IEx) models were used.

**Figure 4:**
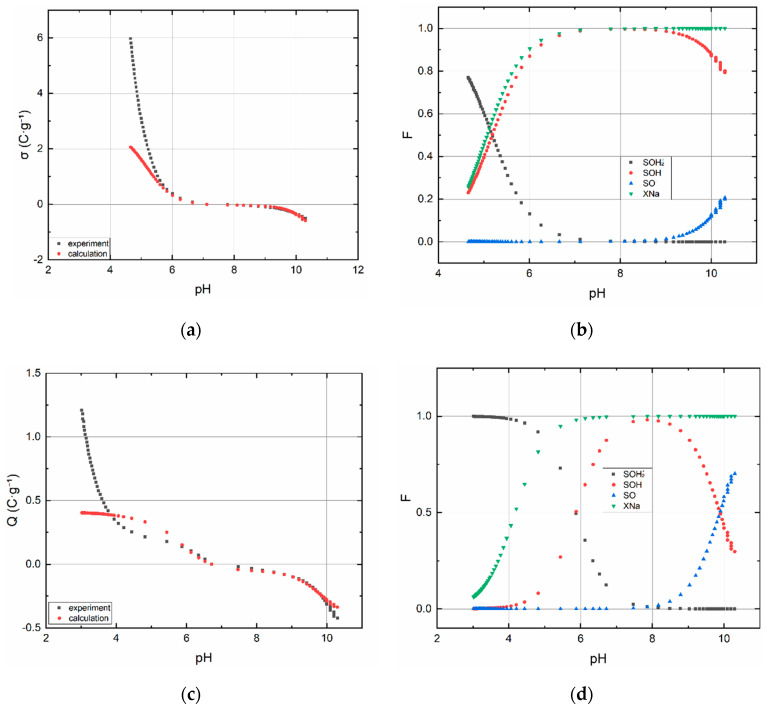
Surface charge dependence on the pH of BC2a (**a**) and BC2b (**c**). Species’ mole fraction dependencies on the pH of BC2a (**b**) and BC2b (**d**). CEM and IEX models were used.

The acid–base titration curve of BC1 and speciation diagram of surface sites are shown in Figure 3a,b, respectively. Sample BC2b containing montmorillonite was prepared specifically for titration, it was not used in other types of experiments. The point of zero charge of BC1 corresponds to pH ∼6.7. The acid–base titration curve of BC2a, BC2b and speciation their diagrams of surface sites are shown in Figure 4a–d. The point of zero charge of BC2a lies around pH ∼7.5 and for BC2b ∼6.6.

Point of zero charge values of BC are extremely volatile depending on preparation and post-treatment. For example, for nonbamboo biochar, these values are as follows, orange peel (9.52), coconut shell (7.98) and golden shower pod (6.65) [38].

Estimated values and input data into code P46DNLRG.fm are displayed in Table 1. The values of the calculated quantities summarized in Table 2 must be assessed with regard to the expected fulfillment of the assumptions under which the CEM and IExM models were derived. In this respect, the relatively most useful data and dependencies were obtained for the sorbent BC2b. In comparison these results with the data and dependencies for with the data for BC2a, these indicate the effect of sample pretreatment, i.e., the effect of removing carbonates, or substances soluble in weakly acidic media in general. This effect is evident from the comparison of the dependencies in Figure 4, especially Figure 4b,d. While the dependencies on Figure 4d have the normal course known for montmorillonite, the dependencies on 4b are completely atypical. This must be attributed to the pretreatment process. In this sense, all quantities obtained for BC1 and BC2a should be considered as indicative and no great weight should be given to their values, despite the fact that WSOS/DF values are acceptable in all cases.

From the point of view of the sorption of anionic components in general, i.e., in our case TcO_4_^−^ and ReO_4_^−^, the most significant dependence is ≡SOH_2_^+^ as a function of pH (see Figure 4b,d and Figure 3b, as well). From this, it is evident that the most suitable is the acidic pH range (2–4), in which practically only ≡SOH_2_^+^ and also the anionic forms Tc(VII) and Re(VII) exist. In other words, in this pH range, the conditions are most favorable for their sorption.

Currently, there is no analytical technique that can distinguish functional groups between edge sites and layer sites in biochar samples. On the other hand, the results of the titration procedure indicate that there are groups that react with acid and caustic soda, i.e., that they are dissociable and that they have a pH-dependent surface charge. A model based on the assumption of the existence of two types of surface groups mentioned below was used to describe the titration curve mentioned. Based on the criterion of fit (WSOS/DF), it can be stated that this model describes the properties of surface groups very well and can be supposed that the two types of functional groups actually exist. It can be assumed that the functional groups (sites) found in the FTIR spectra are located both at the “edges and in the layers” of biochar structure. However, concerning clay minerals, e.g., montmorillonite, have a pH-dependent surface charge distribution, too. For example, approximately at the neutral pH, layer sites are charged negatively while edge sites have zero charge, eventually also partly positive (depends on the values of protonation constants) [39,40]. Therefore, it can be claimed that depending on the pH value, edge sites and/or layer sites of BC2a’s montmorillonite particles affect the sorption of TcO_4_^−^ at pH less than four, more specifically on ≡SiOH_2_^+^ and ≡AlOH^+^ [41].

### 3.2. Batch Adsorption Experiments

#### 3.2.1. Influence of pH

**Figure 5:**
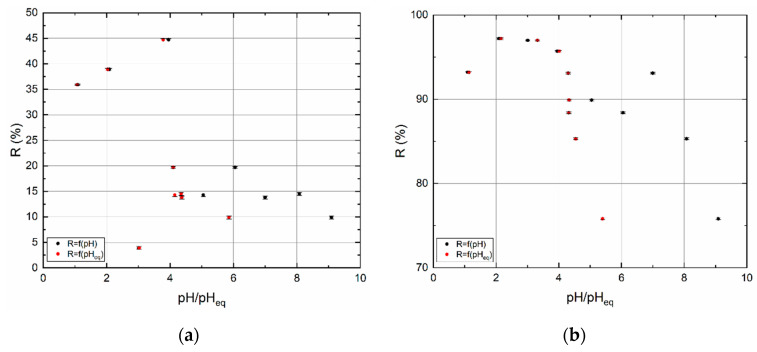
Sorption percentage (*R*) as a function of pH or pH_eq._ (**a**) BC1, and (**b**) BC2a. *t* = 24 h.

The basic batch experiments were performed under conditions found experimentally. The pH of the solution is an important aspect that affects the solid/liquid system as was mentioned above. It involves the surface charges, chemical speciation, etc. The dependence of adsorption percentage on the pH represents Figure 5. Black points correspond to pH of stock solutions (before batch) and red points express equilibrium pH_eq_ of aqueous phase after batch sorption. As can be seen, both samples in alkaline pH do acidification of aqueous phase. For BC1, stock pH 9.09 decreased to pH 5.86, stock pH 8.08 decreased to 4.35 and stock pH 6.05 decreased to 4.15. For BC2a, stock pH 9.09 fell to 5.40, from the stock pH 8.08 to 4.54, and from 6.05 to 4.32. Such a simple comparison implies that surface charge before the pH batch study is positive and depends on the pH of the environment. The highest *R* (45%) for BC1 was reached at pH 4 and for BC2a at pH 2, *R* = 97%. Based on this, it can be seen that the acidic environment acts in favor of ^99m^TcO_4_^−^ (and ReO_4_^−^) sorption.

The excess of H^+^ in solution caused a reduction of surface negative charge in both samples. The increase in the number of positively charged sites was caused by the increasing pH in the system up to pH∼4 (BC1) and pH∼2 (BC2a). Then, the number of active sites decreases rapidly with increasing pH. The adsorption of anions onto carbon materials with naturally, positively charged surface sites (see ≡SOH_2_^+^ above), is due to electrostatic interaction [42], more specifically, due to the anion exchange taking place on the ≡SOH_2_^+^ groups.

#### 3.2.2. Influence of Contact Time

**Figure 6:**
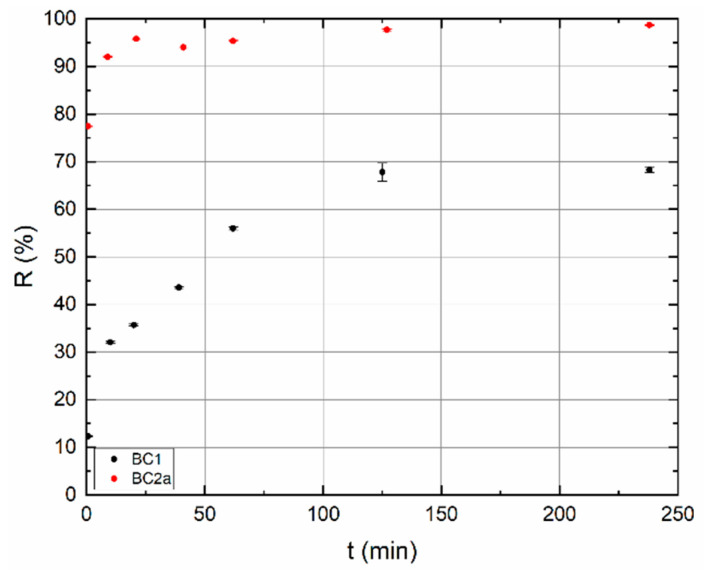
Dependence of sorption percentage (*R*) on contact time (*t*). BC1 pH = 4, BC2a pH = 2.

At the pH values obtained in Section 3.2.1, the influence of contact time was studied. Experimental results are demonstrated in Figure 6. On the BC1, sorption processes are significantly slower than on BC2a. However, the sorption of TcO_4_^−^ is not considered to be different. The rate of sorption depends on a number of parameters, especially on the type of sorbent, the size of the specific surface area, the availability of functional groups, temperature, etc. The results confirm that the sorbents BC1 and BC2a are not identical in these respects. Sorption percentage of BC2a jumped to >92% in 10 min, whereas the sorption percentage of BC1 reached less than 70% in 250 min, in 10 min it is only approx. 30%.

#### 3.2.3. Equilibrium Study

**Figure 7:**
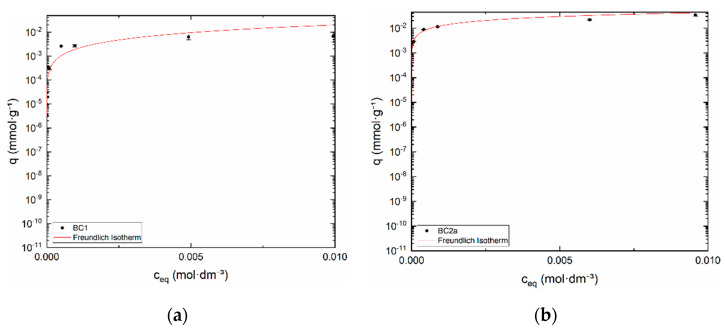
Freundlich isotherm of (**a**) BC1 and (**b**) BC2a. Semilogarithmic scale.

Sorption equilibrium of ^99m^TcO_4_^−^, more exactly of ReO_4_^−^ on BC samples was investigated by batch method. Experimental data were fitted by Freundlich isotherm, see Figure 7a,b. Empirical constants *k_f,S_* and *n_f,S_* are in Table 3 below. Values of weight distribution ratios (*D_g_*) of BC samples reached ≈ 2 × 10^3^ mL·g^−1^. *D_g_* values normally lie between 1 and 10,000 mL·g^−1^.

### 3.3. Dynamic Sorption Experiments

**Figure 8:**
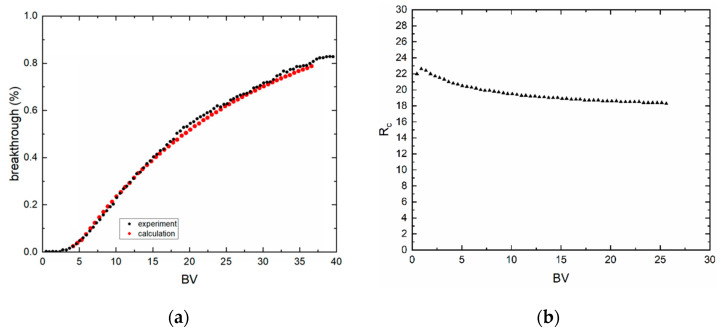
Breakthrough curve of (**a**) BC1, 0.3437 g, pH 2, *c* (NH_4_ReO_4_) = 10^−3^ mol·dm^−3^, *u* = 4.5 BV·h^−1^. (**b**) Retardation coefficient (*R_c_*) as a function of BV.

**Figure 9:**
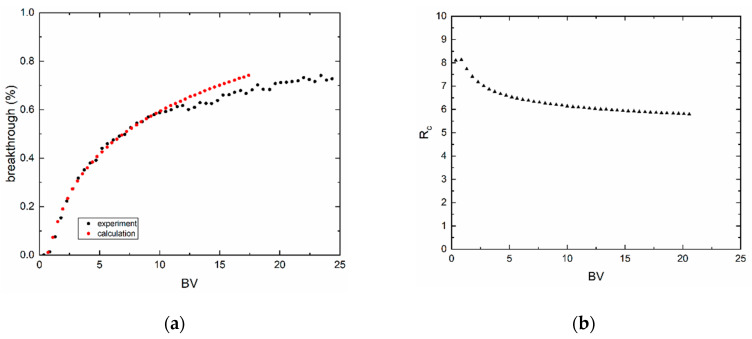
Breakthrough curve of (**a**) BC2a, *m* = 0.431 g, pH 4, *c* (NH_4_ReO_4_) = 10^−4^ mol·dm^−3^, *u* = 4.5 BV·h^−1^. (**b**) Retardation coefficient (*R_c_*) as a function of BV.

Column experiments are plotted as relative count rate (breakthrough, %) as a function of bed volume (*BV*). The relative count rate was calculated as a count rate ratio of sample aliquot and feed aliquot. As can be seen from Figure 8 and Figure 9, breakthrough curves are nonsymmetrical and did not reach 100%. These experiments were limited in time because of the half-life of technetium. Furthermore, sorption processes took a long time due to low surface area. Because of this, practical capacity (*q_m_*) could not be calculated. However, the transport model based on the erfc function could be applied to the evaluation of experimental data. During this, the parameters of Freundlich equilibrium isotherm, *k_f,s_* and *n_f,s_*, and Peclet number (*P_e_*), were sought. The software Famulus 3.5 (Charles University, Prague, Czech Republic) [22,24] and in-house computer code PNLRPA12.fm were used. Fit criterion WSOS/DF is equal to 3.01 × 10^−1^ (BC1) and 3.83 × 10^−2^ (BC2a) and the condition is WSOS/DF ≤ 20. Evidently, the goodness-of-fit is very good, which indicates that the model used corresponds to real conditions. Retardation coefficients (*R_c_*) as a function of BV are not constant (see Figure 8b and Figure 9b) because the isotherms are nonlinear. The values of sought model parameters and of the criteria of goodness-of-fit are in Table 4.

## 4. Conclusions

Although technetium is a man-made element, it occurs in nature in extremely low concentrations, caused by the spontaneous fission of ^238^U. Elevated concentrations of Tc in the environment have been accidentally introduced by the nuclear facility leaks. The most common and environmentally mobile TcO_4_^−^ species need to be immobilized. Biochar, BC1, and biochar/montmorillonite composite, BC2a, represent such a possibility of TcO_4_^−^ separation. Separation and preconcentration are essential in measuring low concentrations of Tc e.g., by AMS method.

Evaluation of potentiometric acid–base titration of sorbents BC1, BC2a, and BC2b and application of CEM and IExM models, allowed to find the mechanism of ^99m^TcO_4_^−^, and ReO_4_^−^ sorption as the carrier, in the range of pH 2–3. Under these conditions, the edge-site functional groups are fully protonated, i.e., have a positive charge and are therefore suitable for capturing negatively charged anionic forms of Tc (VII) and Re (VII).

Computing dynamic model based on erfc function was modified by nonlinear Freundlich equilibrium isotherm and applied for TcO_4_^−^/ReO_4_^−^ sorption on fixed bed plain biochar and biochar/montmorillonite composite. The best fit of dynamic experimental data with calculated one indicates that the computing dynamic model corresponds to real conditions, which were confirmed by the WSOS/DF values.

Regarding the modification of BC in general, it helps to design biochar to target specific functions e.g., reusability, separation purposes, etc. In the respect of BC1 and BC2a properties, it is clear that the composite with montmorillonite K10, BC2a, is more advantageous for the sorption of Tc(VII), especially with regard to faster kinetics and higher sorption capacity. It seems that BC can be effectively used for securing anionic radioactive pollutants as TcO_4_^−^.

## Figures and Tables

**Table 1:** Estimated and inserted parameters as input data into code. These are estimates necessary when using a nonlinear regression procedure.

Sample	log (K_1_)	log (K_2_)	log (K_ex_)	[SOH]_tot_ (mol·kg^−1^)	[S]_tot_ (mol·kg^−1^)	I (mol·dm^−3^)
BC1	15	10	5	0.1	0.1	0.1
BC2a	10	6	5	1	0.5	0.1
BC2b	10	6	3	0.4	0.06	0.1

**Table 2:** Model parameters—calculations.

	**K_1_**	**K_2_**	**K_ex_**	**[SOH]_tot_** (**mol·kg^−1^**)
BC1	(1.54 ± 24.9) × 10^15^	(1.53 ± 0.16) × 10^11^	(8.71 ± 13.8) × 10^−1^	(6.07 ± 0.03) × 10^−2^
BC2a	(9.54 ± 0.15) × 10^10^	(1.97 ± 0.22) × 10^5^	(1.06 ± 11.0) × 10^4^	(2.69 ± 0.35) × 10^0^
BC2b	(9.94 ± 0.79) × 10⁹	(9.60 ± 0.25) × 10^5^	(1.49 ± 0.44) × 10^3^	(4.06 ± 0.54) × 10^−1^
	**WSOS/DF**	**χ^2^**	**σ_i_**	**[S]_tot_** (**mol·kg^−1^**)
BC1	10.3	1.34 × 10^2^	1 × 10^−1^	(8.94 ± 0.05) × 10^−2^
BC2a	17.5	1.15 × 10^3^	1 × 10^−1^	(3.07 ± 8.91) × 10^−2^
BC2b	15.4	9.06 × 10^2^	1 × 10^−1^	(5.04 ± 0.77) × 10^−2^

**Table 3:** Estimated and inserted parameters as input data into code. These are the estimates necessary when using a nonlinear regression procedure.

**Sample**	**k_f,S_**	**P_e_**	**n_f,S_**	**R_c_**	**L** (**cm**)	**u** (**cm·h^−1^**)
BC1	100	5	1	100	2.1	0.185
BC2a	1	1	20	10	2.1	0.223
**Sample**	**c (ReO_4_^−^) (mol·dm^−3^)**	**ε (cm^3^·cm^−3^)**	**ξ (g·cm^−3^)**	-		
BC1	0.001	0.877	0.351			
BC2a	0.0001	0.752	0.323			

**Table 4:** The calculated values of sought model parameters and criteria of goodness-of-fit.

	**k_f,S_**	**P_e_**	**n_f,S_**
BC1	(1.95 ± 0.17) × 10^1^	(1.93 ± 0.06) × 10^0^	(8.71 ± 0.14) × 10^−1^
BC2a	(5.69 ± 0.53) × 10^−1^	(3.65 ± 0.03) × 10^−1^	(6.41 ± 0.11) × 10^−1^
	**WSOS/DF**	**χ^2^**	**σ_i_**
BC1	0.301	16	0.1
BC2a	0.004	1.53	0.1

## References

[1] Li D., Seaman J.C., Kaplan D.I., Heald S.M., Sun C. (2019). Pertechnetate (TcO_4_−) sequestration from groundwater by cost-effective organoclays and granular activated carbon under oxic-environmental conditions. Chem. Eng. J..

[2] Hu H., Jiang B., Wu H., Zhang J., Chen X.H. (2016). Bamboo (acidosasa edulis) shoot shell biochar: Its potential isolation and mechanism to perrhenate as a chemical surrogate for pertechnetate. J. Environ. Radioac..

[3] Rajec P., Rosskopfova O., Galamboš M., Frišták V., Soja G., Dafnomili A., Noli F., Ðukicć A., Matović L.J. (2016). Sorption and desorption of pertechnetate on biochar under static batch and dynamic conditions. J. Radioanal. Nucl. Chem..

[4] Chen L., Yin X., Yu Q., Lu S., Meng F., Ning S., Wang X., Wei Y. (2019). Rapid and selective capture of perrhenate anion from simulated groundwater by a mesoporous silica-supported anion exchanger. Microporous Mesoporous Mater..

[5] Nicholson S., Sanders T.W., Blaine L.M. (1993). The determination of low levels of ^99^Tc in environmental samples by inductively coupled plasma-mass spectrometry. Sci. Total. Environ..

[6] Chen Q., Dahlgaard H., Hansen H.J.M., Aarkrog A. (1990). Determination of ^99^Tc in environmental samples by anion exchange and liquid-liquid extraction at controlled valency. Anal. Chim. Acta.

[7] Attrep M., Enochs J.A., Broz L.D. (1971). Atmospheric technetium-99. Environ. Sci. Technol..

[8] Serne R.J., Crum J.V., Riley B.J., Levitskaia T.G. (2016). Options for the Separation and Immobilization of Technetium.

[9] TrisKem International Extraction Chromatography. https://www.triskem-international.com/scripts/files/5addcf96423962.97324869/technical_doc_all-products_web-0.pdf.

[10] Viglašová E., Daňo M., Galamboš M., Rosskopfová O., Rajec P., Novák I. (2016). Column studies for the separation of ^99^mTc using activated carbon. J. Radioanal. Nucl. Chem..

[11] Shi K., Hou X., Roos P., Wu W. (2012). Determination of technetium-99 in environmental samples: A review. Anal. Chim. Acta.

[12] Cornett R.J., Zhao X.-L., Hou X.-L., Kieser W.E. (2019). A preliminary study of ^99^Tc measurement using matrix-assisted low energy AMS. Nucl. Instrum. Methods Phys. Res. Sec. B.

[13] Bergquist B.A., Marchetti A.A., Martinelli R.E., McAninch J.E., Nimz G.J., Proctor I.D., Southon J.R., Vogel J.S. (2000). Technetium measurements by accelerator mass spectrometry at LLNL. Nucl. Instrum. Methods Phys. Res. Sec. B.

[14] Povinec P. (2011). Analysis of Environmental Radionuclides.

[15] Triskem TEVA Resin, Product Sheet. https://www.triskem-international.com/scripts/files/5c5855b887c4f4.23796223/PS_TEVA-Resin_EN_160927.pdf.

[16] Eichrom Analytical Procedure Technetium-99 in Water. https://www.eichrom.com/eichrom/methods/eichrom-methods/.

[17] Viglašová E., Galamboš M., Dankovaá Z., Krivosudský L., Lengauer C.L., Hood-Nowotny R., Soja G., Rompel A., Matík M., Briančin J. (2018). Production, characterization and adsorption studies of bamboo-based biochar/montmorillonite composite for nitrate removal. Waste Manag..

[18] Chacón F.J., Sánchez-Monedero M.A., Lezama L., Cayuela M.L. (2020). Enhancing biochar redox properties through feedstock selection, metal preloading and post-pyrolysis treatments. Chem. Eng. J..

[19] Wu L., Jing S.Z., Ding X.W. (2020). Phosphorus retention using iron (II/III) modified biochar in saline-alkaline soils: Adsorption, column and field tests. Environ. Pollut..

[20] Pandey D., Daverey A., Arunachalam K. (2020). Biochar: Production, properties and emerging role as a support for enzyme immobilization. J. Clean. Prod..

[21] Khan M.B., Cui X., Jilan G., Lu L.T.M., Cao X., Sahito Z.A., Hamid Y., Hussain B., Yang X., He Z. (2020). New insight into the impact of biochar during vermi-stabilization of divergent biowastes: Literature synthesis and research pursuits. Chemosphere.

[22] Filipská H., Štamberg K. (2005). Mathematical modeling of a Cs(I)-Sr(II)-bentonite-magnetite sorption system, simulating the processes taking place in a deep geological repository. Acta Polytech..

[23] Wanner H., Albinsson Y., Karnland O., Wieland E., Wersin P., Charlet L. (1994). The acid-base chemistry of montmorillonite. Radiochim. Acta.

[24] Dvořák L., Ledvinka M., Sobotka M. (1993). Famulus 3.5. Software.

[25] Rahmani A., Mousavi H.Z., Fazli M. (2010). Effect of nanostructure alumina on adsorption of heavy metals. Desalination.

[26] Palágyi Š., Štamberg K. (2010). Modeling of transport of radionuclides in beds of crushed crystalline rocks under equilibrium non-linear sorption isotherm conditions. Radiochim. Acta.

[27] Palágyi Š., Štamberg K., Vopálka D. (2017). Simplified modeling in dynamic column technique for the determination of radionuclide transport parameters in systems of solid granular materials and groundwater. J. Radioanal. Nucl. Chem..

[28] Štamberg K., Palágyi Š. (2011). Effect of grain size on the sorption and desorption of 137Cs in crushed granite columns and groundwater system under dynamic conditions. J. Radioanal. Nucl. Chem..

[29] Nartey O.D., Zhao B. (2014). Biochar preparation, characterization, and adsorptive capacity and its effect on bioavailability of contaminants: An overview. Adv. Matter. Sci. Eng..

[30] Batista E.M.C.C., Shultz J., Matos T.T.S., Fornari M.R., Ferreira T.M., Szpoganicz B., De Freitas R.A., Mangrich A.S. (2018). Effect of surface and porosity of biochar on water holding capacity aiming indirectly at preservation of the Amazon biome. Sci. Rep..

[31] Wu M., Feng Q., Sun X., Wang H., Gielen G., Wu W. (2015). Rice (Oryza sativa L) plantation affects the stability of biochar in paddy soil. Sci. Rep..

[32] Liu Y., He Z., Uchimiya M. (2015). Comparison of biochar formation from various agricultural by-products using FTIR spectroscopy. Mod. Appl. Sci..

[33] Zhao J., Shen X.-J., Domene X., Alcañiz J.-M., Liao X., Palet C. (2019). Comparison of biochars derived from diferent types of feedstock and their potential for heavy metal removal in multiple-metal solutions. Sci. Rep..

[34] IBI (International Biochar Iniciative) Standardized Product Definition and Product Testing Guidelines for Biochar That Is Used in Soil. https://biochar-international.org/wp-content/uploads/2020/06/IBI_Biochar_Standards_V2.1_Final2.pdf.

[35] Wijitkosum S., Jiwnok P. (2019). Elemental composition of biochar obtained from agricultural waste for soil amendment and carbon sequestration. Appl. Sci..

[36] Waqas M., Aburiazaiza A., Miandad R., Rehan M., Barakat M., Nizami A.-S. (2018). Development of biochar as fuel and catalyst in energy recovery technologies. J. Clean. Prod..

[37] Manna S., Singh N., Purakayastha T., Berns A.E.E. (2020). Effect of deashing on physico-chemical properties of wheat and rice straw biochars and potential sorption of pyrazosulfuron-ethyl. Arab. J. Chem..

[38] Sen T.K. (2017). Point of Zero Charge and Effect of Solution pH. Air, Gas, and Water Pollution Control Using Industrial and Agricultural Solid Waste Adsorbents.

[39] Guo Y., Yu X. (2017). Characterizing the surface charge of clay minerals with Atomic Force Microscope (AFM). AIMS Mater. Sci..

[40] Liu J., Gaikwad R., Hande A., Das S., Thundat T. (2015). Mapping and quantifying surface charges on clay nanoparticles. Langmuir.

[41] Liu X., Lu X., Sprik M., Cheng J., Meijer E.J., Wang R. (2013). Acidity of edge surface sites of montmorillonite and kaolinite. Geochim. Cosmochim. Acta.

[42] Demiral H., Gündüzoğlu G. (2010). Removal of nitrate from aqueous solutions by activated carbon prepared from sugar beet bagasse. Bioresour. Technol..

